# 1,8-Cineol (Eucalyptol) Disrupts Membrane Integrity and Induces Oxidative Stress in Methicillin-Resistant *Staphylococcus aureus*

**DOI:** 10.3390/antiox12071388

**Published:** 2023-07-06

**Authors:** Abderrahmen Merghni, Ahmed Reda Belmamoun, Adriana Cristina Urcan, Otilia Bobiş, Mohamed Ali Lassoued

**Affiliations:** 1 Laboratory of Antimicrobial Resistance LR99ES09, Faculty of Medicine of Tunis, University of Tunis El Manar, Tunis 1007, Tunisia; 2 Department of Agricultural Sciences, Faculty of Nature and Life Sciences, Djillali Liabes University, Sidi-Bel-Abbes 22000, Algeria; vetsba@gmail.com; 3 Department of Microbiology and Immunology, Faculty of Animal Science and Biotechnology, University of Agricultural Sciences and Veterinary Medicine Cluj-Napoca, 400372 Cluj-Napoca, Romania; adriana.urcan@usamvcluj.ro; 4 Department of Beekeeping and Sericulture, Faculty of Animal Science and Biotechnology, University of Agricultural Sciences and Veterinary Medicine Cluj-Napoca, 400372 Cluj-Napoca, Romania; 5 Laboratory of Pharmaceutical, Chemical and Pharmacological Drug Development LR12ES09, Faculty of Phamacy, University of Monastir, Monastir 5000, Tunisia; lassoued98@yahoo.fr

**Keywords:** 1,8-cineol, MRSA, membrane integrity, oxidative stress, ROS, MDA, antioxidant enzymes

## Abstract

Due to the increased emergence of drug-resistant bacteria, the declining efficiency of traditional antimicrobials has generated severe concerns in recent years. Subsequently, more interest in other antimicrobial agents from natural resources draws more attention as an alternative to conventional medications. This study investigated the bactericidal mechanism of monoterpene 1,8-cineol (eucalyptol), a major compound of various essential oils, against methicillin-resistant *Staphylococcus aureus* (MRSA). The antibacterial activity of 1,8-cineol was assessed by an MTT assay against clinical and reference MRSA strains. A cell membrane integrity test, followed by zeta potential (ZP) measurements, was performed to evaluate the disruption of the bacterial membrane integrity. Additionally, the cytotoxic effect of this molecule on MRSA bacteria was investigated by monitoring reactive oxygen species (ROS) generation, lipid peroxidation (MDA), and antioxidant enzyme activities (CAT and SOD). Regarding the anti-staphylococcal effect, the obtained results revealed the antibacterial efficacy of 1,8-cineol wherein the minimum inhibitory concentrations were equal to 7.23 mg/mL. Furthermore, it enhanced membrane permeability, with a 5.36-fold increase in nucleic acid and protein leakage as compared with untreated strains, along with the alteration of surface charge (ZP) in MRSA cells. The tested compound caused an increase in ROS generation reaching 17,462 FU and MDA production, reaching 9.56 μM/mg protein, in treated bacterial cells, along with a decrease in oxidative stress enzymes activities. Our findings suggest that 1,8-cineol has the ability to damage the membrane integrity and induce ROS-mediated oxidative stress in MRSA cells, leading to its antagonistic effect against this pathogen and consequently aiding in the reversal of antibiotic resistance.

## 1. Introduction

*Staphylococcus aureus*, one of the Gram-positive opportunistic pathogens, is frequently a major source of hospital-acquired illnesses, including bloodstream infections, skin and soft tissue infections [1], lung infections and cystic fibrosis [2], and implant-related infections [3], especially in vulnerable patients such as the immunocompromised and older adults. Methicillin-resistant *S. aureus* (MRSA) infection is one of the most prevalent causes of hospital-acquired infections, and it is frequently linked with high mortality, morbidity, and financial burden [4]. Previously associated with health care settings, MRSA has emerged as a significant source of community-associated illnesses, creating reservoirs in both contexts [5].

Antimicrobial resistance is still one of the most serious concerns threatening human health across the world. *S. aureus* is one of the major causes of nosocomial infections by Gram-positive bacteria [6]. It is highly susceptible to penicillin and a wide range of other antibiotics [7]. Due to indiscriminate use, strains of *S. aureus* have developed resistance to many routinely used antimicrobials, such as penicillin, which is mediated by the production of β-lactamase enzyme. Furthermore, MRSA strains have emerged with antimicrobial resistance to all penicillins, including methicillin and nearly all β-lactam antimicrobials [8].

Natural products originating from plants have attracted interest because of their possible involvement in reducing antibiotic resistance [9]. For instance, essential oils extracted from aromatic and medicinal plants offer enormous antibacterial potential due to their efficient bactericidal activity against a wide range of pathogenic bacteria [10].

Essential oils are a complex molecular combination that can contain up to 300 distinct components in varying proportions [11]. They are distinguished by two or three major components, primarily monoterpenes with relatively high concentrations (20–70%), while the other constituents are present in minor proportions [12]. Volatile compounds of EOs, produced by aromatic and medicinal plants as secondary metabolites, are derived from terpenoids, mainly monoterpenes, and have been identified as hydrocarbons and their oxygenated derivatives in the form of phenols, alcohols, ethers, esters, aldehydes, peroxides, and ketones. In addition, there are non-terpenoid compounds, namely, phenylpropanoids, fatty acids, and their esters, and, less frequently, nitrogen- and sulfur-containing compounds [13]. The biological activities of essential oils are mostly attributed to their main components. However, it is critical to note that the aforementioned properties are a result of the synergistic interaction between minor and major components [14]. The mechanism causing the cell death of pathogenic bacteria can be attributed to the interaction of these bioactive substances with lipid bilayers, perturbation of plasma membrane functionality [15], and loss of cellular membrane integrity [16]. Another mode of action of plant antimicrobials is destabilizing the biofilm matrix, inhibition of bacterial enzymes and respiratory activity, reduction of the ATP levels, depolarization of the membrane potential, and inhibition of the nucleic acids transcription [17].

Commonly known as eucalyptol, 1,8-cineol is a bicyclic monoterpene that occurs in several essential oils of various plants such as *Eucalyptus globulus*, *Rosmarinus officinalis*, *Origanum vulgare*, *Thymus vulgaris*, and *Coriandrum sativum* [12,13,18,19]. Monoterpenes, such as 1,8-cineol, exhibit strong antibacterial activities against a wide range of drug-resistant pathogenic bacteria [14,20]. These compounds affect bacterial cell permeability and enhance membrane fluidity, resulting in a change in the topology of membrane proteins and a stoppage in the cell respiratory process [15,21]. Moreover, their antibacterial mechanism is associated with enhancement of reactive oxygen species (ROS) in the exposed cells, which induce oxidative stress and consequently inhibit certain essential biological processes [6,9]. The present study was undertaken to investigate 1,8-cineol’s antibacterial potency against MRSA strains, as well as its effects on bacterial membrane integrity, reactive oxygen species generation, and lipid peroxidation. The impact of the tested chemical on the production of oxidative stress enzymes was also studied.

## 2. Materials and Methods

### 2.1. Chemical and Bacterial Strains

The chemical component 1,8-cineol (99% purity), also known as eucalyptol, was purchased commercially from a local manufacturer (Parachimic, Sfax, Tunisia). Regarding bacterial strains, two strains of MRSA were used in this study: a reference strain of *Staphylococcus aureus* ATCC 43300 obtained from American Type Culture (Manassas, VA, USA) and a clinical isolate (Sa15) previously characterized by our research group [22]. This strain harbored the *mecA* gene and was high biofilm forming. To ensure optimal bacterial development, the MRSA strains were sub-cultured twice in BHI broth and incubated at 37 °C for 24 h before each use.

### 2.2. Antibacterial Activity of 1,8-Cineol

The antibacterial activity was assessed by the determination of the minimum inhibitory concentration (MIC) and minimum bactericidal concentration (MBC) values. The MICs of 1,8-cineol against MRSA strains were evaluated by the broth dilution method as previously described [23]. Overnight cultures (37 °C) of tested strains in Muller Hinton (MH) broth medium were prepared by adjusting the turbidity of each bacterial culture to achieve an optical density of 0.5 McFarland (McF) standards. The broth dilution method was carried out in a 96-well microtiter plate. The 1,8-cineol was prepared aseptically, and two-fold serial dilutions in dimethylsulfoxide (DMSO) were transferred to sterile 96-well microtiter plates (190 μL per well) and then diluted in MH broth. Finally, the inocula (10 μL) of each strain (0.5 McF) were added to each well. A few wells were reserved for sterility control (no inoculum) and inoculum viability (no compound). After incubation for 24 h at 37 °C, the bacterial growth inhibition was measured by adding 20 μL of MTT (Methyl thiazolyldiphenyl tetrazolium bromide, Sigma-Aldrich, St. Louis, MI, USA). The formation of purple colored formazan indicated viable cells, while the yellow color implied bacterial death. The lowest concentration of the samples that visually inhibited the bacterial growth was considered as the MIC [23].

To determine the MBC values, 20 μL of each well medium with no visible growth was removed and plated in MH agar. After 24 h of incubation at 37 °C, the MBC was defined as the lowest concentration at which 99% of the bacteria were killed.

The MBC/MIC ratio was determined to estimate the antibacterial potency of the examined 1,8-cineol. When this ratio was less than or equal to 4, the EO was considered bactericidal; however, it was called bacteriostatic when the ratio was higher than 4 [24].

### 2.3. Effects on Membrane Permeability

The effect of 1,8-cineol on the cell membrane integrity of MRSA strains was evaluated by monitoring the leakage of cellular contents as previously described [25]. Overnight bacterial cultures of ATCC and the clinical isolate were washed with PBS and re-suspended in 0.8% saline solution (OD_600nm_ = 0.4). The strains were treated with 1,8-cineol (MIC) for 2 h, while the untreated strains served as positive controls. After incubation at 37 °C, the supernatants from bacterial cell suspensions were collected by centrifugation at 3000 rpm for 10 min. Then, the membrane integrity was determined by quantifying the release of cellular constituents by recording the absorbance spectrophotometry at 260 nm to detect the nucleotide leakage, while the absorbance at 280 nm was used to detect the presence of proteins.

### 2.4. Surface Charge Alteration

The relation between the Zeta potential (ZP) and the physiological state of the bacteria has been conveniently employed to characterize the damage to the bacterial structure as a result of different stressors since conserved ZP values correlated with the preserved structures of surface macromolecules and with the physiological state of the cells [26]. Here we determined ZP to study the interaction of 1,8-cineol with the bacterial envelope. Overnight MRSA cultures in MH agar were diluted (OD_600nm_ = 0.11) in MH broth. Then 1,8-cineol was added in different concentrations (MIC/2; MIC, MIC × 2, MIC × 4) to the bacteria suspensions and incubated for 2 h or 24 h. Afterward, the suspension was washed with PBS three times, and the ZP values were measured using a Zetasizer Nano-S (Malvern^®^Instruments, Malvern, UK) at 25 °C [27]. The results of the ZP were expressed in millivolts (mV), and the measurements were performed in triplicate and averaged.

### 2.5. Reactive Oxygen Species Generation

The production of reactive oxygen species (ROS) by MRSA cells treated with 1,8-cineol was determined using a peroxynitrite indicator, 20–70-dichlorodihydrofluorescein diacetate (DCFH-DA) (SigmaAldrich, Gillingham, UK). The adjusted bacterial cultures (0.5 McF) were treated with different concentrations of 1,8-cineol (corresponding to MIC, MIC × 2, and MIC × 4) in the presence of DCFH-DA at a final concentration of 5 mM in 0.85% saline and incubated at 37 °C aerobically for 24 h. The untreated bacterial culture served as a negative control. The fluorescence emission of DCFH-DA was measured at 525 nm using a Tecan microtiter plate reader with an excitation wavelength of 485 nm [28]. The background fluorescence of 0.85% saline and autofluorescence of the bacterial cells incubated without the probe were measured to calculate the net fluorescence emitted from the assay itself. The experiment was conducted in triplicate. The amount of ROS produced intracellularly was proportional to the intensity of DCF fluorescence [29]. The used concentrations were standardized using H_2_O_2_ as a positive control. The experiment was conducted in triplicate.

### 2.6. Lipid Peroxidation

The production of malondialdehyde (MDA), a commonly used marker for oxidative stress, was quantified in MRSA cells exposed to 1,8-cineol. Briefly, the adjusted bacterial cultures (0.5 McF) were treated with different concentrations of 1,8-cineol corresponding to one, two, and four times the MIC at 37 °C aerobically, whereas the control was incubated with 0.85% (*w*/*v*) saline alone for 24 h. One hundred mL of the SDS lysis solution was added to an 100 mL aliquot of the treated culture and incubated for 5 min at room temperature. The mixtures were then incubated at 95 °C for 60 min in presence of a thiobarbituric acid (TBA) reagent. Each of the mixtures was cooled to room temperature in an ice bath for 5 min and centrifuged at 3000× *g* for 15 min. The supernatants were then collected, and the absorbances were read at 532 nm. The concentrations of MDA in each treatment were calculated based on the standard curve of absorbance against MDA concentration (ranged from 0, 10, 20, 30, 40, to 50 µmol/L). This assay was performed in triplicates [30].

### 2.7. Antioxidant Enzyme Activity

For the determination of superoxide dismutase (SOD) and catalase (CAT) enzyme activities, MRSA (1 × 10^8^ CFU/mL, 500 μL) cultures from the late exponential growth phase were treated with various concentrations of 1,8-cineol (MIC, MIC × 2, and MIC × 4) and incubated for 24 h at 37 °C. The suspension was centrifuged at 3000 rpm for 10 min to aspirate the supernatant, and the resultant pellet was washed twice with PBS and re-suspended in 500 μL of cell lysate buffer (10 Mm Tris-HCl, 1 mM EDTA, 0.1% Triton-X100 and 150 mM NaCl) and kept for incubation at 37 °C for 1 h. The contents were then centrifuged at 3000 rpm for 10 min, and the supernatant was collected for enzyme assays [31].

#### 2.7.1. Measurement of Catalase (CAT) Enzyme Activity

CAT activity in the bacterial cell lysate was measured as previously described [32]. Briefly, in a quartz cuvette, 780 µL of phosphate buffer (KH_2_PO_2_/K_2_HPO_4_, pH 7) were introduced with 200 µL H_2_O_2_ (20 mM), to which 20 µL of bacterial protein extract was added. The optical density of the mixture in each cell was monitored for 60 s (t = 0 s and =60 s) at a wavelength of 240 nm. One unit (U) of enzyme activity was defined as the amount of enzyme required to convert 1 µmol of H_2_O_2_ in one second.

#### 2.7.2. Measurement of Superoxide Dismutase (SOD) Enzyme Activity

The SOD activity in the bacterial cell lysate was analyzed based on the ability of this enzyme to inhibit the anti-oxidation of pyrogallol at 420 nm. A volume of 0.1 mL of each bacterial extract was incubated with 2.85 mL of Tris HCl and 25 µL of pyrogallol for 30 s. A unit of SOD activity was defined as the amount of enzyme that inhibited the rate of pyrogallol oxidation [33]. Then the activity of the SOD was measured at 420 nm as follows: % inhibition = (blank Abs − Abs test)/Abs test.

### 2.8. Statistical Analysis

All the experiments were performed in triplicate, and the obtained data were presented as means ± standard deviations. The data were further analyzed using the one-way analysis of variance (ANOVA) test followed by Tukey’s post hoc test to calculate the significance of the results. *p* values less than 0.05 were considered significantly statistically different.

## 3. Results

### 3.1. Antibacterial Activity

The antibacterial effects of the 1,8-cineol are reported as “in vitro” activity as MIC and MBC and summarized in Table 1. The tested molecule exerted a bacteriostatic effect against both MRSA strains, with MIC values equal to 7.23 mg/mL. The MBC values against *S. aureus* ATCC 43300 and Sa15 were found to be equal to 57.87 mg/mL and 115.75 mg/mL, respectively. As the ratio MBC/MIC > 4, the effect of 1,8-cineol against both MRSA strains was considered as bacteriostatic [24].

### 3.2. Effects on Membrane Permeability

**Figure 1:**
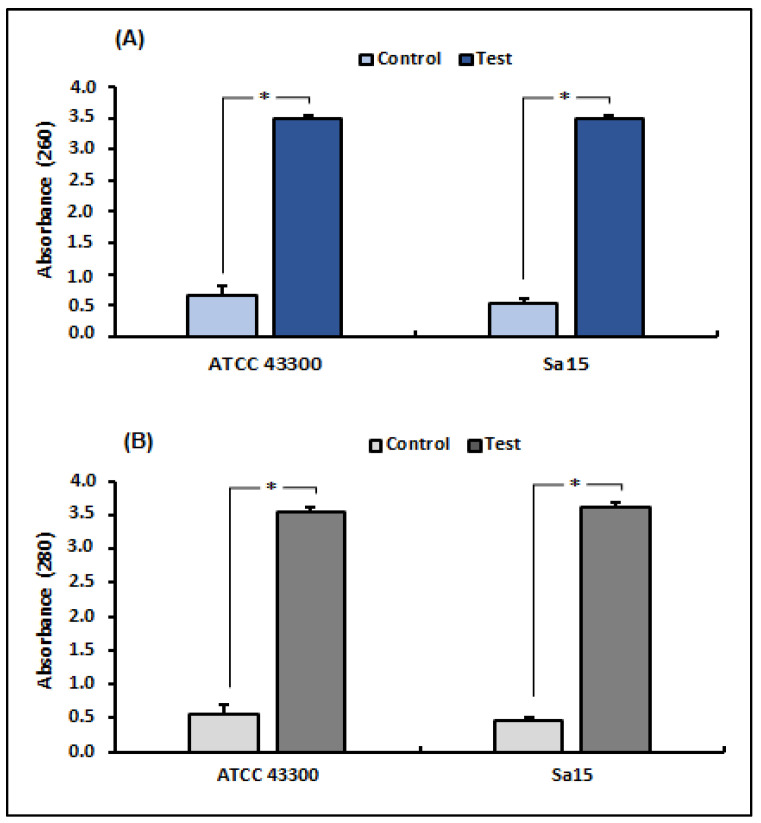
Release of bacterial cell content, assessed by measuring the absorbance at 260 nm (**A**) and 280 nm (**B**), in both MRSA strains (ATCC 43300 and Sa15) treated with 1,8-cineol. The results are expressed as mean absorbance ± SD. * Represents significant difference (*p* < 0.05) between each treated strain with the negative control.

UV-VS spectrophotometry was used to measure nucleotide and protein leakage in MRSA strains to assess the effect of 1,8-cineol on bacterial membrane integrity (Figure 1). Our results revealed a significant increase (*p* < 0.05) in supernatant absorbance due to the release of nucleic acids (260 nm) and proteins (280 nm) by tested strains treated with 1,8-cineol, compared with the control (untreated strains). Exposure to this molecule (MICs) showed an increase in absorbance from 5.36 times (ATCC strain) to 6.58 times (Sa15) at 260 nm and from 6.25 (ATCC strain) to 8 (Sa15) times at 280 nm, compared with the untreated *S. aureus* strains.

### 3.3. Surface Charge Alteration

ZP measurement is one of the important parameters for examining the impact of antibacterial substances on the surface of bacteria. The surface charge of bacterial cells, treated with 1,8-cineol, was assessed based on their zeta potential value (Table 2). Under no-stress conditions, MRSA cells became more negatively charged with the increasing age of the culture: between −13.5 ± 3.4 and −27.1 ± 1 mV during the 24 h of the assay. Bacterial cells exposed to 1,8-cineol had larger negative zeta potential values than non-stressed cells at each tested period (2 h and 24 h), with the values growing more negative with the concentration-dependent manner of the tested molecule. Interestingly, the zeta potential increased significantly from −18.5 mV to −27.4 mV (*p* < 0.05) after exposing the MRSA reference strain to various concentrations of test compounds for 2 h, whereas no significant changes were registered after 24 h of treatment (*p* > 0.05). In clinical strain Sa15, a more pronounced increase in the negative charge from 2.07-fold (MIC/2) to 2.92-fold (MIC × 4) was observed after 2 h of treatment with 1,8-cineol. Similarly, the test compounds increased the zeta potential of the Sa15 strain more than the 43300 strain, after 24 h of treatment.

### 3.4. Generation of Reactive Oxygen Species

**Figure 2:**
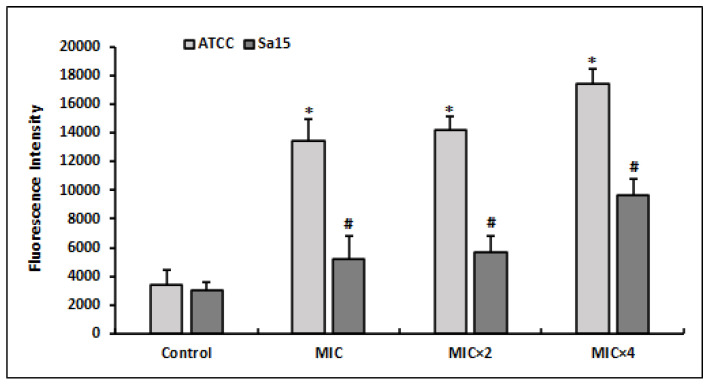
Quantitation of intracellular ROS production by MRSA strains (ATCC 43300 and Sa15) after 24 h treatment with various concentrations of 1,8-cineol (MIC to MIC × 4), using the DCFA-DA probe. The results are expressed as the mean fluorescence intensity ± SD. * and # represent significant difference (*p* < 0.05) between each treatment with the negative control.

The DCFH-DA indicator was used to measure ROS generation by MRSA strains treated with various doses of l,8-cineol (Figure 2). ROS production was found to be increased in bacterial cells subjected to different doses of l,8-cineol (MIC to MIC × 4) when compared with the control (untreated cells). Additionally, the effect of the tested chemical was more pronounced on the reference strain ATCC 43300, with an increase in ROS generation (reaching 17,462 FU), when compared with the clinical strain Sa15. Overall, the effect of l,8-cineol on ROS generation was dosage dependent in both MRSA strains.

### 3.5. Lipid Peroxidation

**Figure 3:**
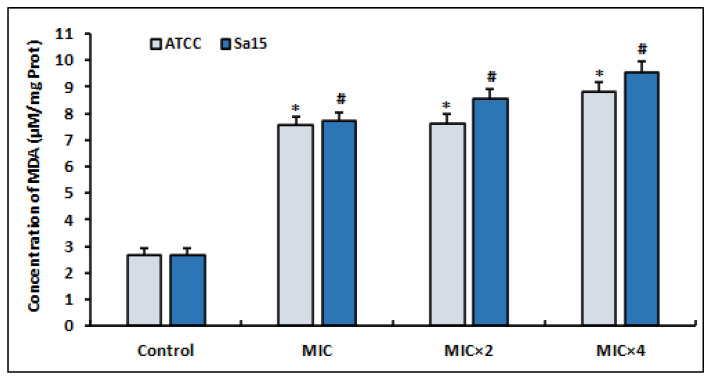
Lipid peroxidation induced in MRSA cells (ATCC 43300 and Sa15), incubated for 24 h with various concentrations of 1,8-cineol (MIC to MIC × 4) and measured by the production of malondialdehyde (MDA). The data are expressed as the mean ± SD of three independent experiments. Values are significantly different (*; # *p* < 0.05) compared with the negative control.

The results of the MDA levels, a commonly used marker of oxidative stress, produced by MRSA strains treated with l,8-cineol are shown in Figure 3. After 24 h of treatment with various concentrations of the tested agent (MIC to MIC × 4), the detected MDA levels increased significantly in both bacterial strains compared with the untreated control (*p* < 0.05). Interestingly, the increased production of ROS in the treated bacterial cells (Figure 2) caused the enhancement of lipid peroxidation.

### 3.6. Antioxidant Enzyme Activity

**Figure 4:**
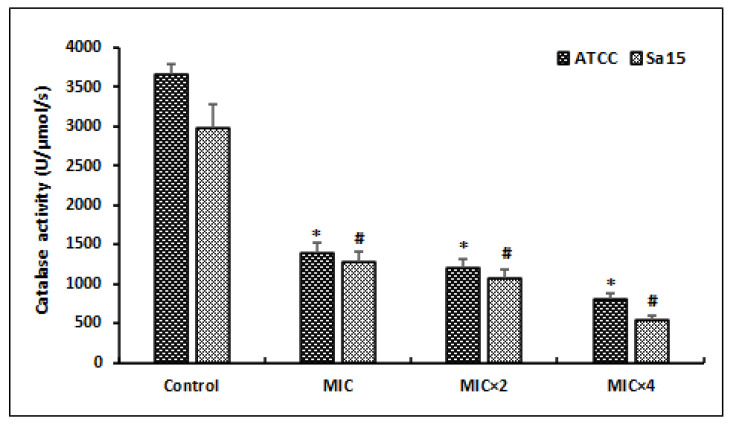
Effect of various concentrations of l,8-cineol on the antioxidant enzyme activity (catalase) in MRSA cells (ATCC 43300 and Sa15). The strains were incubated with different concentrations of l,8-cineol (MIC to MIC × 4) for 24 h. *; # represents a significant difference (*p* < 0.05) between each treatment and the negative control.

After treating MRSA cells with different doses of l,8-cineol during 24 h, the catalase activity (CAT) was determined (Figure 4).

Our results revealed that several doses of MIC of l,8-cineol exhibited a significant decrease in this anti-oxidant enzyme activity (CAT), in a dose-dependent manner. The lowest CAT activities in the reference and clinical strains, corresponding to 812 ± 81 U/mg protein and 544 ± 54 U/mg protein, respectively, were registered at a high concentration (MIC × 4) of tested agent (*p* < 0.05).

**Figure 5:**
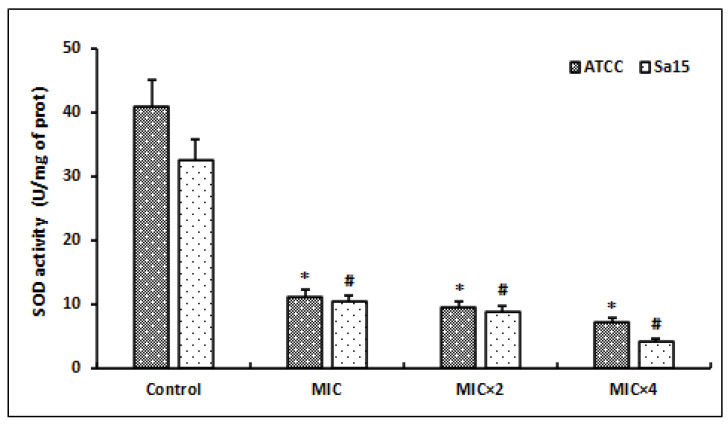
Effect of various concentrations of l,8-cineol on the antioxidant enzyme activity (superoxide dismutase) in MRSA cells (ATCC 43300 and Sa15). The strains were incubated with different concentrations of l,8-cineol (MIC to MIC × 4) for 24 h. *; # represent a significant difference (*p* < 0.05) between each treatment and the negative control.

Following the MRSA strains’ exposure to different concentrations of l,8-cineol, we evaluated the superoxide dismutase (SOD) activity after 24 h of incubation (Figure 5).

The SOD levels were significantly decreased in the bacterial lysates of the tested strains after treatment with this molecule (*p* < 0.05). Initially, the untreated bacterial cells (control) showed SOD amounts of 32.6 to 41.1 U/mg proteins in the ATCC and Sa15 strains, respectively. The SOD values were significantly decreased after the treatment with doses of MIC and MIC × 2 (*p* < 0.05). Higher concentrations of this molecule (MIC × 4) further decreased the SOD levels to 4.2 U/mg and 7.2 U/mg protein in the Sa15 and ATCC 43300 strains, respectively.

## 4. Discussion

The investigation of new antimicrobial molecules, as an alternative to antibiotics, based on biologically active non-toxic compounds, has increased significantly during the last decade [34]. Secondary metabolites derivatives from plants such as monoterpenes are well known for their antimicrobial potential [35]. For instance, l,8-cineol is a cyclic oxygenated monoterpene with potent anti-staphylococcal effects [36,37]. To fight against pathogenic bacteria using biological methods, we investigated the anti-MRSA activities of l,8-cineol. Our results showed that the tested molecule exerted a bacteriostatic effect against clinical and reference strains (Sa15 and ATCC 43300), with an MIC value equal to 7.23 mg/mL. This finding was in agreement with previous reports showing the effusiveness of l,8-cineol against MRSA and methicillin-sensitive *S. aureus* (MSSA) strains [21,22,23,24,25,27,28,29,30,31,32,33,34,35,36,37,38,39]. Additionally, it was reported that the eucalyptol was active against a wide range of pathogenic strains with various values of MICs [40]. Due to its small size and non-polar structure, 1,8-cineol is a biofilm-penetrating chemical with recognized antibacterial potential, interestingly, against *S. aureus* bacterium [41].

The antimicrobial efficacy of this chemical component depends on a variety of criteria such as the tested specie, the inoculum and the culture medium, the mechanism of action of the tested agent, etc. [42].

In the second part of our investigation, we evaluated the effect of l,8-cineol in the membrane permeability and bacterial surface charge of treated cells. Our data indicated that the tested component (different MICs) induced cell membrane damage in both MRSA strains, resulting in cytoplasmic content release. In fact, the l,8-cineol induced leakage of proteins and nucleic acids, which consequently reflected a loss of permeability and integrity of the bacterial membrane [43]. Regarding the bacterial surface charge, we highlight the value of ZP measurements to explore the effect of the l,8-cineol on the bacterial cell surface. This relationship between the physiological bacterial state and the zeta potential has proved useful in characterizing the damage to the bacterial structure as a result of various stressors [26]. Our finding revealed that the bacterial cells exposed to the l,8-cineol presented zeta potential values more negative than those of non-treated cells. This was in agreement with previous reports showing that treated *S. aureus* cells exhibited a higher negative net surface charge (more negative ZP) as the *S. aureus* cultures aged (exposure duration) and the antibiotic concentration increased [44]. Similarly, it was reported that Thymol was toxic for treated *S. aureus*, with increased capacity to enhance cell surface charge and to elicit intracellular materials leakage [45].

Most Gram-positive bacteria have a negative ZP, which is likely due to the prevalence of negatively charged functional groups on their surface such as peptidoglycan and teichoic and lipoteichoic acids [46]. Numerous investigations have found that the conserved ZP values correspond with surface macromolecule structure preservation and cell physiological condition [47,48].

The induction of oxidative stress in MRSA strains treated with different doses of l,8-cineol was assessed by detecting reactive oxygen species (ROS) generation. Our results revealed that the tested compound induced a dose-dependent increase in the formation of ROS as compared with the control. It was recently demonstrated that exposing *S. aureus* to different essential oils concentrations led to the accumulation of ROS, which was followed by cell death due to post-stress ROS-mediated toxicity [49]. Additionally, it was previously reported that l,8-cineol causes oxidative stress in carbapenemase-producing *Klebsiella pneumoniae* cells by generating ROS [9]. The increased ROS accumulation in treated cells disrupts essential biological processes and damages nucleic acids, proteins, and lipids, which consequently inhibit bacterial growth [50]. Another indicator of the stress condition in the treated cells is defined by the level of malondialdehyde (MDA) [10]. Both MRSA strains exposed to l,8-cineol showed higher concentrations of MDA than the untreated cells, reflecting the presence of lipid peroxidation caused by this molecule as a result of increased intracellular ROS generation, which ultimately led to cell death [30]. It was previously shown that terpene chemicals from many plants produce ROS, which attack membrane lipids, triggering a chain reaction that eventually disrupts the bacterial membrane [51].

To survive, numerous bacterial species produce the enzyme “catalase,” which aids in cell detoxication and allows them to repair or escape the oxidative damage caused by hydrogen peroxide [52]. The reduction in catalase activity in MRSA cells after treatment with l,8-cineol has also been demonstrated with other phytochemicals such as Silibine, which has reduced this enzyme activity and caused toxicity in *S. aureus* [53]. Similarly, a recent study on the anti-staphylococcal activity of catechin revealed a decrease in catalase activity in MRSA and MSSA after exposure to different MICs of this compound, compared with untreated cells [54]. In addition to catalase, superoxyde dismutase, which catalyzes the dismutation of superoxyde into hydrogen peroxyde, represents the first line of defense for bacterial cells against ROS. Similar to our results, methanolic extract from *Andrographis paniculata* showed a 0.7-fold decrease in SOD activity in treated *S. aureus* cells compared with untreated cells [55]. SOD is vital for oxidant defense because it makes *S. aureus* more resistant to oxidative stress [56]. *S. aureus* produces two major SODs, SOD-A and SOD-M. The first is involved in endogenous stress, whereas the second is involved in exogenous stress [57]. The suppression of SOD activity results in a decrease in the conversion of O_2_^−^ to H_2_O_2_, which likely results in an increase in O_2_^−^ levels and leads to the toxicity of *S. aureus* cells [58].

## 5. Conclusions

Our study investigated the antibacterial efficacy and the mode of action of 1,8-cineol against MRSA strains. This compound exhibited potent antibacterial activity, with alterations of bacterial surface charge. Additionally, 1,8-cineol induced oxidative stress in tested strains, leading to bacterial membrane disruption via intracellular material leakage and lipid peroxidation. Moreover, the influx of generated ROS affected the antioxidant enzyme activity and attacked macromolecules causing bacterial damage and consequently cell death. Our finding highlights the antibacterial potentialities of 1,8-cineol and suggests its valorization for the development of new anti-infective agents.

## Figures and Tables

**Table 1:** Antibacterial activity of 1,8-cineol against MRSA strains.

MRSA Strain	Analyzed Parameter	Concentration (mg/mL)
43300 *	MIC	7.23
	MBC	57.87
	MBC/MIC (ratio)	8
Sa15 **	MIC	7.23
	MBC	115.75
	MBC/MIC (ratio)	16

* 43300: reference strain (ATCC); ** Sa 15: clinical isolate. MIC—minimum inhibitory concentration; MBC—minimum bactericidal concentration.

**Table 2:** Net surface charge of MRSA cells during exposure to 1,8-cineol.

Strains	Period	Control	MIC/2	MIC	MIC × 2	MIC × 4
43300 *	2 h	−18.5 ±0.3	−27.4 ± 0.7 ***	−27.5 ± 0.7 ***	−27.5 ± 0.5 ***	−27.1 ± 0.4 ***
	24 h	−27.1 ± 1.0	−27.8 ± 0.3	−28.1 ± 1.0	−29.2 ± 0.6 ***	−30.2 ± 0.7 ***
Sa15 **	2 h	−13.5 ± 3.4	−28 ± 1.0 ***	−35.9 ± 1.4 ***	−37.0 ± 2.5 ***	−39.5 ± 1.1 ***
	24 h	−21.9 ± 0.8	−31.8 ± 1.3 ***	−36.7 ± 0.6 ***	−37.4 ± 2.7 ***	−43.5 ± 0.5 ***

* 43300: reference strain (ATCC); ** Sa 15: clinical isolate; ***: significant difference (*p* < 0.05) compared with the control. MIC: minimum inhibitory concentration.

## Data Availability

Not applicable.
